# Differential acclimation kinetics of the two forms of type IV chromatic acclimaters occurring in marine *Synechococcus* cyanobacteria

**DOI:** 10.3389/fmicb.2024.1349322

**Published:** 2024-02-16

**Authors:** Louison Dufour, Laurence Garczarek, Bastian Gouriou, Julia Clairet, Morgane Ratin, Frédéric Partensky

**Affiliations:** Sorbonne Université, CNRS, UMR 7144 Adaptation and Diversity in the Marine Environment (AD2M), Station Biologique de Roscoff (SBR), Roscoff, France

**Keywords:** marine picocyanobacteria, *Synechococcus*, chromatic acclimation, spectral niche, phycobilisome, comparative physiology

## Abstract

*Synechococcus*, the second most abundant marine phytoplanktonic organism, displays the widest variety of pigment content of all marine oxyphototrophs, explaining its ability to colonize all spectral niches occurring in the upper lit layer of oceans. Seven *Synechococcus* pigment types (PTs) have been described so far based on the phycobiliprotein composition and chromophorylation of their light-harvesting complexes, called phycobilisomes. The most elaborate and abundant PT (3d) in the open ocean consists of cells capable of type IV chromatic acclimation (CA4), i.e., to reversibly modify the ratio of the blue light-absorbing phycourobilin (PUB) to the green light-absorbing phycoerythrobilin (PEB) in phycobilisome rods to match the ambient light color. Two genetically distinct types of chromatic acclimaters, so-called PTs 3dA and 3dB, occur at similar global abundance in the ocean, but the precise physiological differences between these two types and the reasons for their complementary niche partitioning in the field remain obscure. Here, photoacclimation experiments in different mixes of blue and green light of representatives of these two PTs demonstrated that they differ by the ratio of blue-to-green light required to trigger the CA4 process. Furthermore, shift experiments between 100% blue and 100% green light, and *vice-versa*, revealed significant discrepancies between the acclimation pace of the two types of chromatic acclimaters. This study provides novel insights into the finely tuned adaptation mechanisms used by *Synechococcus* cells to colonize the whole underwater light field.

## Introduction

Phytoplanktonic cells have an obligate requirement for light to perform photosynthesis. Yet in the marine environment, this energy source is highly variable quantitatively and qualitatively with depth but also along coast-offshore gradients, leaving aside daily oscillations (Kirk, 1994; Holtrop et al., 2021). This variability has triggered an extensive structural and pigment diversification of phytoplankton light-harvesting antennae, which enable cells to considerably enhance the wavelength range they can collect and therefore the number of photons reaching photosystems. Despite their apparent simplicity compared to algae and higher plants, cyanobacteria possess the most elaborated form of antennae known in oxyphototrophs, called phycobilisomes (PBS). PBS are huge water-soluble complexes composed of six to eight rods radiating around a central core. Both core and rods are constituted of phycobiliproteins that bind open-chain tetrapyrroles, called phycobilins (Sidler, 1994). While the PBS core is always made of allophycocyanin and is highly conserved, PBS rods display a very large structural flexibility since they can be made of phycocyanin (PC) only, or of PC and one or two phycoerythrin (PE) types, PE-I and PE-II (Ong and Glazer, 1991; Six et al., 2007). Additionally, each phycobiliprotein can bind up to three different kinds of phycobilins. The ultimate degree of sophistication is the capacity for some cyanobacterial cells to modify the composition of their PBS in response to changes in the ambient light color. This process called “chromatic acclimation” (CA)—the initial term was actually “chromatic adaptation,” but was recently replaced in order to best describe this physiological process (Shukla et al., 2012)—was first observed in the early 20th century in freshwater cyanobacteria shifted from red to green light (Engelmann, 1902; Gaidukov, 1903). CA was later on attributed to changes in the phycobiliprotein composition of PBS rods: in red light, rods are entirely composed of PC and cells look green, whereas in green light PC is restricted to the base of the rods and the distal part is made of PE, causing cells to exhibit a bright red color (Boresch, 1922). This type of complementary chromatic acclimation (CCA, also called type 3 chromatic acclimation or CA3) is one among the seven different types of CA known so far in cyanobacteria (Tandeau de Marsac, 1977; Hirose et al., 2019; Sanfilippo et al., 2019a). While CA2—a simple type of CA where PE production is induced in green light, generating longer PBS rods, and repressed in red light—and CA3 occur mainly in freshwater and brackish cyanobacteria, CA4 is the main type occurring in the open ocean and is specific to marine *Synechococcus* cyanobacteria (Palenik, 2001; Everroad et al., 2006; Humily et al., 2013). Contrary to CA2 and CA3, CA4 does not involve changes in the phycobiliprotein composition of PBS rods, but in their phycobilin composition. In response to shifts between green light (GL) and blue light (BL), chromatic acclimaters can indeed modify the relative amount of the two phycobilins bound to PE-I and PE-II in order to match the predominant ambient light color. More specifically, they exhibit in BL a high ratio of the BL-absorbing phycourobilin (PUB, *λ*_max_ ≈ 495 nm) to the GL-absorbing phycoerythrobilin (PEB, *λ*_max_ ≈ 545 nm), and *vice-versa* in GL (Everroad et al., 2006; Shukla et al., 2012). Variations in PUB and PEB cell content are generally assessed by measuring the relative ratio of whole cell fluorescence excitation at 495 and 545 nm (Exc_495:545_) with emission set at 580 nm, which in chromatic acclimaters changes from 0.6–0.7 in GL to 1.6–1.7 in BL (Palenik, 2001; Everroad et al., 2006). In the nomenclature of *Synechococcus* pigment types (PTs) established by Six et al. (2007) and later modified by Humily et al. (2013), chromatic acclimaters are classified as “PT 3d” cells, meaning that they possess PBS rods made of PC, PE-I and PE-II, a feature shared by all PT 3 representatives, and can modify their Exc_495:545_ ratio. In contrast, PTs 3a, 3b, and 3c display a constitutively low, medium and high Exc_495:545_ ratio, respectively. For this reason, PT 3a strains are often referred to as “green light specialists” and PT 3c as “blue light specialists” (Grébert et al., 2018, 2022).

Two genetically different types of chromatic acclimaters have been described so far: PTs 3dA and 3dB (Humily et al., 2013). Both possess a small genomic island involved in the CA4 process, yet the CA4-A and CA4-B islands differ genetically and structurally (Humily et al., 2013; Sanfilippo et al., 2019b; Grébert et al., 2021). Although long overlooked, CA4 appears to be an ecologically important process since chromatic acclimaters were shown to account for more than 40% of the whole marine *Synechococcus* population along the *Tara* oceans expedition transect (Grébert et al., 2018). Moreover, PTs 3dA and 3dB were found to be equally abundant (22.6% and 18.9%, respectively) but distributed in complementary ecological niches in the field. The former was indeed predominant in cold, nutrient-rich and highly productive waters at high latitude, as well as in other vertically mixed environments, while the latter was mostly found in nitrogen and phosphorus-poor oceanic areas and appeared to be more abundant at depth. The emergence and maintenance of two CA4 types over the course of evolution, as well as the differential distribution of PTs 3dA and 3dB in the environment (Grébert et al., 2018), strongly suggest that they may not be as phenotypically equivalent as previously thought (Humily et al., 2013). To check this hypothesis, we acclimated three representatives of each PT 3dA and 3dB in batch culture under two conditions of temperature, two light irradiances and five light colors in order to compare their growth rates and PBS properties. Furthermore, we performed shifts from BL to GL (and *vice-versa*) at two irradiances to compare the CA4 kinetics between five strains of each PT. These experiments demonstrated that PT 3dA and 3dB strains actually differ in the blue-to-green light ratio necessary to trigger the CA4 process, and revealed some significant discrepancies in their acclimation pace.

## Materials and methods

### Biological material and culture conditions

Ten *Synechococcus* strains, of which five PT 3dA and five PT 3dB representatives (Table 1), were retrieved from the Roscoff Culture Collection.^1^ These strains were isolated from diverse environments and selected based on their genome availability (Doré et al., 2020) and clade affiliation, in order to include the CA4-A model strain RS9916 (Shukla et al., 2012; Sanfilippo et al., 2016, 2019b) as well as representatives from all five major clades (I to IV and CRD1) in the global ocean (Farrant et al., 2016).

**Table 1:** Characteristics of the different *Synechococcus* strains used in this study.

Strain name	RCC #^a^	Subcluster^b^	Clade^b^	Subclade^c^	Pigment type^d^	Isolation region
BIOS-U3-1	2,533	5.1	CRD1	n.a.	3dA	Chile upwelling
BL107	515	5.1	IV	IVa	3dA	Balearic Sea
MITS9220	2,571	5.1	CRD1	n.a.	3dA	Equatorial Pacific
RS9916	555	5.1	IX	n.a.	3dA	Gulf of Aqaba
WH8020	751	5.1	I	Ia	3dA	Sargasso Sea
A15-62	2,374	5.1	II	IIa	3dB	Off Mauritania
A18-40	n.a.	5.1	III	IIIa	3dB	Atlantic Ocean
MINOS11	2,319	5.3	n.a.	n.a.	3dB	Mediterranean Sea
PROS-U-1	2,369	5.1	II	IIh	3dB	Moroccan upwelling
RS9915	2,553	5.1	III	IIIa	3dB	Gulf of Aqaba

a Roscoff Culture Collection.

b Farrant et al. (2016).

c Mazard et al. (2012).

d Humily et al. (2013).

Cells were grown in 50 mL polystyrene flasks (Sarstedt, Germany) in PCR-S11 medium (Rippka et al., 2000) supplemented with 1 mM sodium nitrate. All were pre-acclimated prior to measurements for at least 3 weeks in continuous light provided by blue and/or green LEDs (Alpheus, France) in temperature-controlled chambers.

### Acclimation experiments

A selection of six out of the 10 abovementioned strains (BL107, RS9916 and WH8020 for PT 3dA and A15-62, PROS-U-1 and RS9915 for PT 3dB; Table 1) were grown in the following conditions: (i) two temperatures: 18°C and 25°C; (ii) two irradiances: low light (LL, 15 μmol photons m^−2^ s^−1^) and high light (HL, 75 μmol photons m^−2^ s^−1^); (iii) five light qualities: blue light (100% BL), green light (100% GL), as well as three mixes of blue-green light: 25% BL–75% GL, 50% BL–50% GL and 75% BL–25% GL. Both temperatures were selected based on Ferrieux et al. (2022), which recently demonstrated that 18 and 25°C were the lowest and highest temperatures at which a selection of *Synechococcus* strains belonging to the five major clades in the environment were capable of growing. The two irradiances were chosen as being similar to those used in a previous study by Humily et al. (2013), for easier comparison of results between the two studies.

The light intensity and visible spectra of LEDs were measured using a PG200N Spectral PAR Meter (UPRtek, Taiwan; Supplementary Figure S1). Each strain was grown in triplicate and inoculated at an initial cell density of 3 × 10^6^ cells mL^−1^. Samples were harvested every day to measure cell concentration and fluorescence parameters by flow cytometry, and once during the exponential phase to measure phycobilin and phycobiliprotein contents by spectrofluorimetry (see below).

### Shift experiments

Shift experiments between 100% low BL (LBL) and 100% low GL (LGL) and *vice-versa*, and between 100% high BL (HBL) and 100% high GL (HGL) and *vice-versa*, were performed on all 10 *Synechococcus* strains mentioned in Table 1, but only at 25°C. Each strain was diluted with fresh medium before the beginning of the experiments and regularly transferred in order to avoid limitation by nutrients. Aliquots were collected two to three times a day, depending on light intensity, to measure phycobilin content by spectrofluorimetry (see below).

### Flow cytometry

Culture aliquots were sampled twice a day, fixed with 0.25% (v/v) glutaraldehyde (grade II, Sigma Aldrich, United States) and stored at −80°C until analysis (Marie et al., 1999). Cell density was determined using a Guava easyCyte flow cytometer equipped with a 488 nm laser and the Guavasoft software (Luminex Corporation, Texas). Average orange (583 nm) and red (695 nm) fluorescence signals were used as proxies of the phycoerythrin (PE) and chlorophyll *a* (Chl *a*) contents per cell, respectively. Both signals were normalized to that of standard fluorescent 0.95 μm silica beads.

### Spectrofluorimetry

*In vivo* fluorescence spectra were recorded at 240 nm min^−1^ with slits fixed at 10 nm once during the exponential phase using a spectrofluorimeter FL6500 (Perkin-Elmer, United-States). Excitation spectra were acquired between 450 and 560 nm with emission set at 580 nm, corresponding to the PE emission maximum. Emission spectra were recorded between 550 and 750 nm with excitation set at 530 nm, close to the PEB excitation maximum. Spectra were monitored and analyzed with the Fluorescence software (Perkin-Elmer). The Exc_495:545_ fluorescence excitation ratio was used as a proxy for the PUB:PEB ratio. The Em_560:650_ and Em_650:680_ fluorescence emission ratios were used as proxies of the PE to PC and PC to PBS terminal acceptor (TA) ratios, respectively. The first parameter provided information about the electron transfer efficiency within the PBS and/or the length of PBS rods, and the second one about the coupling of PBS to PSII reaction center chlorophylls.

### Statistical analyses

All statistical analyses were conducted using the R software (version 4.2.3; R Core Team, 2021) in order to test for significant differences between PTs 3dA and 3dB. The potential influence of growing conditions (temperature, light quality and quantity) was also investigated.

For acclimation experiments, a linear mixed model (nlme package version 3.1-164; Pinheiro et al., 2023) was fit to each variable (growth rate, flow cytometry fluorescence signals, fluorescence excitation and emission ratios), by considering the temperature, light intensity, light color and pigment type as fixed factors, and the strain as a random factor.

For shift experiments, the slopes of the linear parts of Exc_495:545_ vs. time curves were compared between PTs 3dA and 3dB representatives using *t*-tests. As the number of strains used for shift experiments was reduced (*n* = 3 for PT 3dA and *n* = 5 for PT 3dB), the significance level was raised to 0.1 in order to confer more power to statistical analyses.

## Results

### Acclimation experiments

A first set of experiments was performed to investigate the effect of temperature, light intensity and color on various physiological characteristics including growth rate, flow cytometric red and orange fluorescence signals, as well as phycobilin and phycobiliprotein content, of six *Synechococcus* strains (BL107, RS9916 and WH8020 for PT 3dA and A15-62, PROS-U-1 and RS9915 for PT 3dB; Table 1) pre-acclimated for at least 3 weeks to the different tested conditions.

### Growth rate

**Figure 1:**
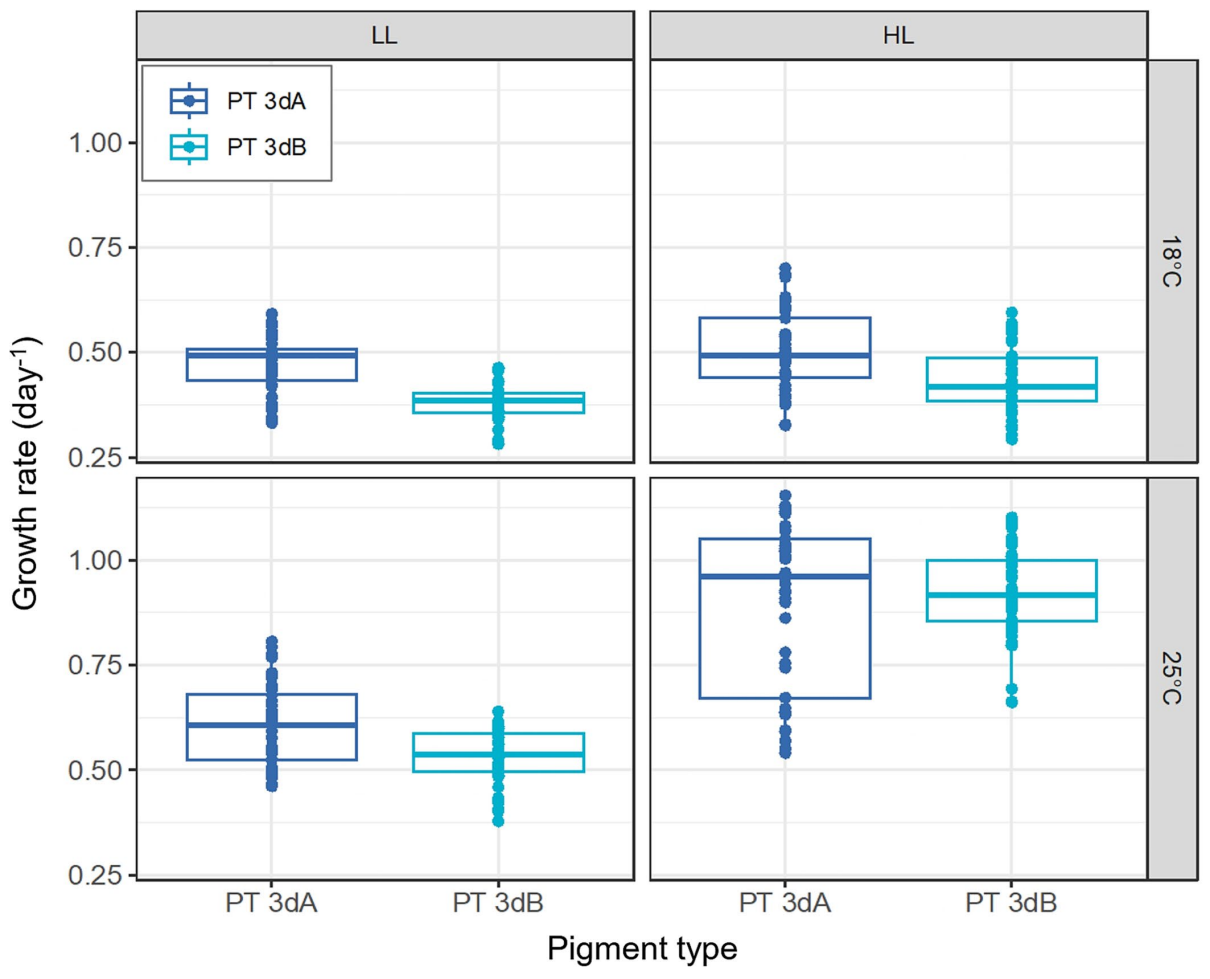
Growth rates of six PT 3dA and 3dB representatives acclimated to the different conditions of temperature and light intensity used for acclimation experiments. Each boxplot represents the measurements performed for all representatives of each pigment type in the five light colors tested in this study (*n* = 45). The light quality factor is not shown, as it had no significant effect on growth rate (*p*-value >0.05). LL, 15 μmol photons m^−2^ s^−1^; HL, 75 μmol photons m^−2^ s^−1^; PT, pigment type.

The growth rate (*μ*) of the six *Synechococcus* strains was lower at 18°C than at 25°C (*p*-value <0.05; Figure 1; Supplementary Table S1). Maximal *μ* values were reached at 25°C in HL, with all strains except WH8020 achieving more than one cell division per day (*μ* > 0.69 day^−1^; Supplementary Figure S2). While a clear increase in growth rates of both PTs was seen between LL and HL at 25°C, a less marked difference was noted at 18°C, suggesting that temperature and light intensity had a synergistic effect on *μ*. This was confirmed by the mixed model, which highlighted an interaction effect between the two factors (*p*-value <0.05; Supplementary Table S1). While for any given strain, the growth rate varied little between the different light colors (Supplementary Figure S2), PT 3dA representatives globally grew faster than their PT 3dB counterparts (*p*-value <0.05; Figure 1; Supplementary Table S1).

### Chlorophyll *a* and phycoerythrin fluorescence

**Figure 2:**
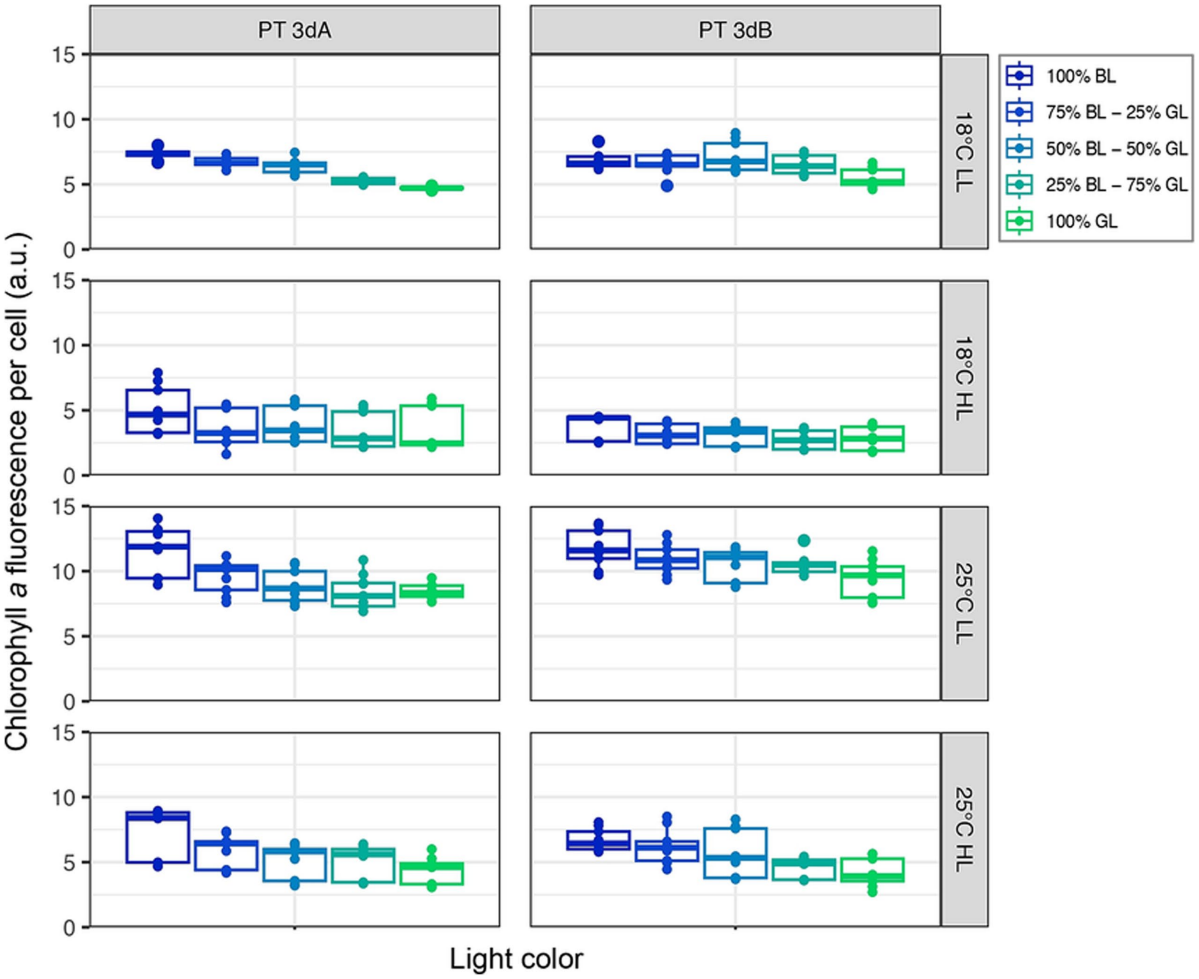
Flow cytometric chlorophyll *a* fluorescence per cell of six PT 3dA and 3dB representatives acclimated to the different conditions of temperature, light intensity and quality used for acclimation experiments. Each boxplot represents the measurements performed for all representatives of each pigment type in one light color condition (*n* = 9). LL, 15 μmol photons m^−2^ s^−1^; HL, 75 μmol photons m^−2^ s^−1^; PT, pigment type; BL, blue light; GL, green light.

**Figure 3:**
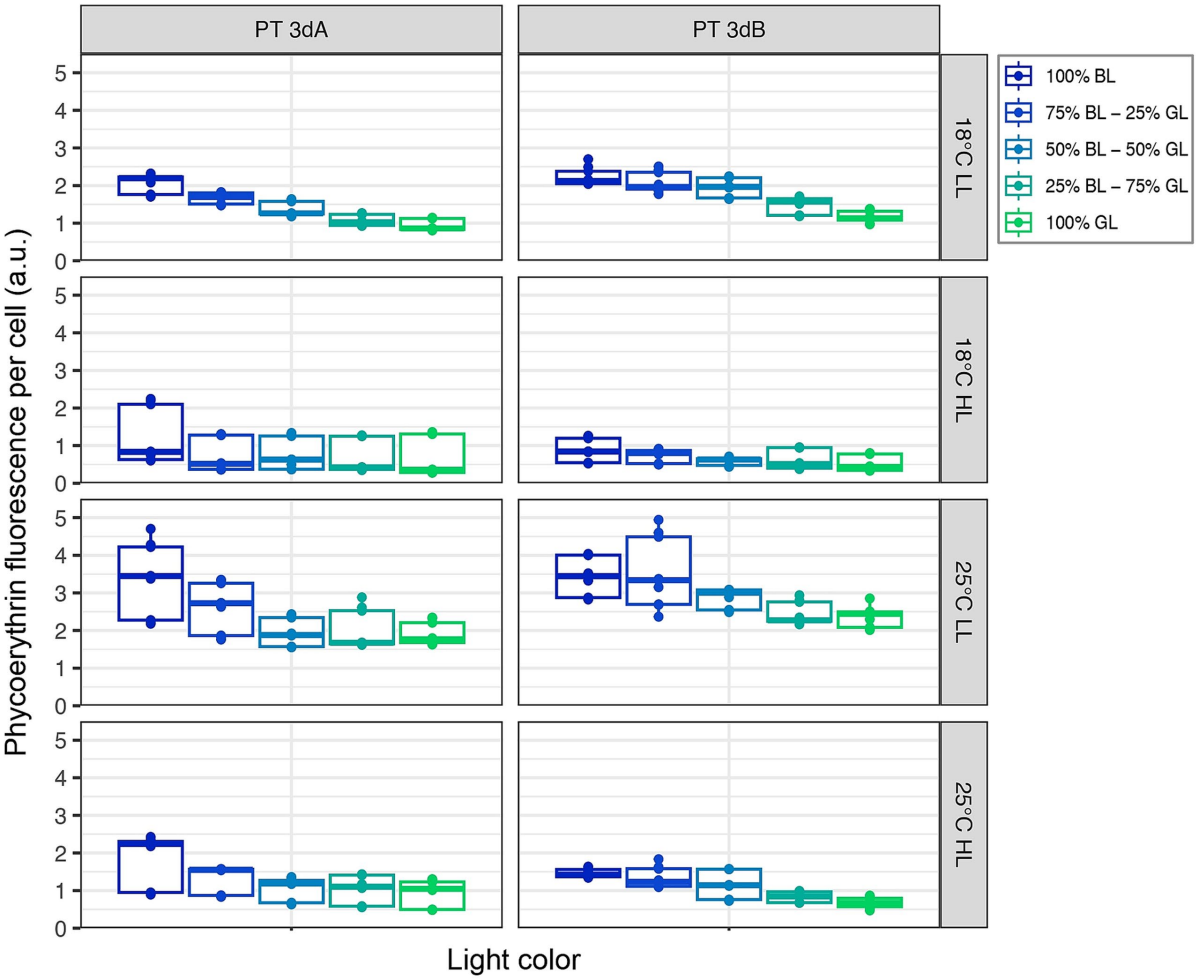
Same as Figure 2 but for the flow cytometric phycoerythrin fluorescence per cell.

A significant downward trend in both flow cytometric red (Chl *a*) and orange (PE) fluorescence signals was observed from 100% BL to 100% GL for both PTs in all conditions (*p*-value <0.05; Figures 2, 3; Supplementary Figures S3, S4; Supplementary Table S1). Besides light quality, temperature and light intensity strongly impacted Chl *a* and PE fluorescence signals (*p*-value <0.05; Supplementary Table S1). Both variables were indeed higher in LL than HL, but also at 25°C compared to 18°C (Figures 2, 3). Due to the synergistic effect of temperature and irradiance (*p*-value <0.05; Supplementary Table S1), maximum values were measured at 25°C in LL, and conversely minima were associated with the 18°C and HL condition.

### Phycobilin content

All strains globally displayed Exc_495:545_ fluorescence excitation ratio, a proxy of the whole cell PUB:PEB ratio, typical of chromatic acclimaters when grown in 100% GL (Exc_495:545_ ≈ 0.6–0.7) or 100% BL (Exc_495:545_ ≈ 1.6–1.7; Humily et al., 2013; Supplementary Figure S5). It is important to note that the LEDs used to get the 100% GL condition actually peaked at 515 nm, which is at the blue edge of the green wavelength range (Supplementary Figure S1). Yet, the fact that all tested strains exhibited the lowest possible Exc_495:545_ ratio for chromatic acclimaters shows that they did sense this light quality as being full GL.

**Figure 4:**
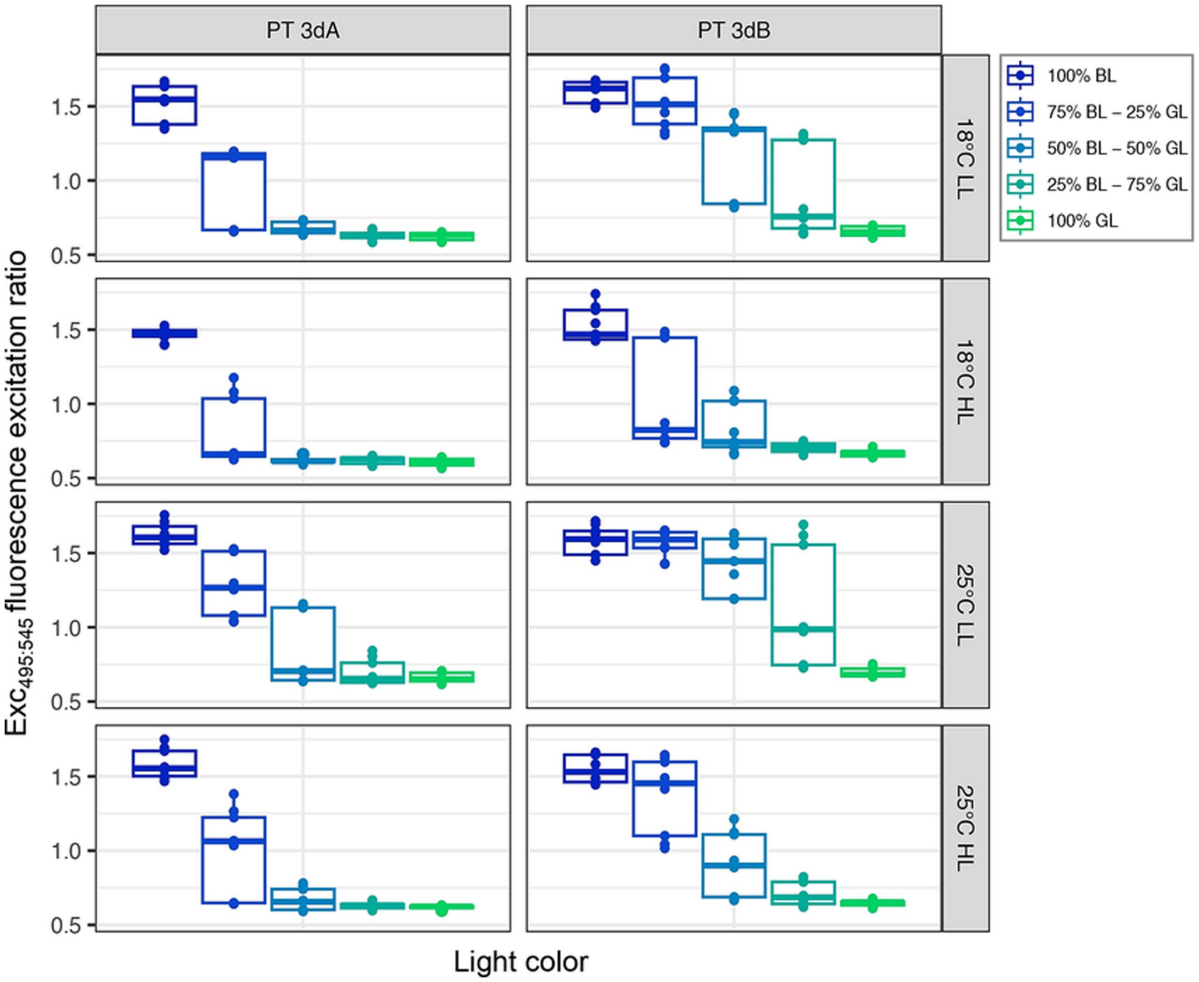
Same as Figure 2 but for the Exc_495:545_ fluorescence excitation ratio, a proxy of the whole cell PUB:PEB ratio.

The light quality had the strongest impact on the Exc_495:545_ fluorescence excitation ratio (*p*-value <0.05; Supplementary Table S1), the latter expectedly decreasing from 100% BL to 100% GL. Interestingly, the two PTs did not respond in the same way to the light color (*p*-value <0.05; Supplementary Table S1). PT 3dB representatives indeed exhibited higher Exc_495:545_ ratios than their PT 3dA counterparts in most intermediate blue-green conditions (Figure 4), due to a more progressive decrease of their ratio from the BL- to the GL-acclimated state. This trend was more pronounced in LL than HL conditions, as confirmed by the significant interaction effect between PT and light quantity (*p*-value <0.05; Supplementary Table S1).

### Phycobiliprotein content

**Figure 5:**
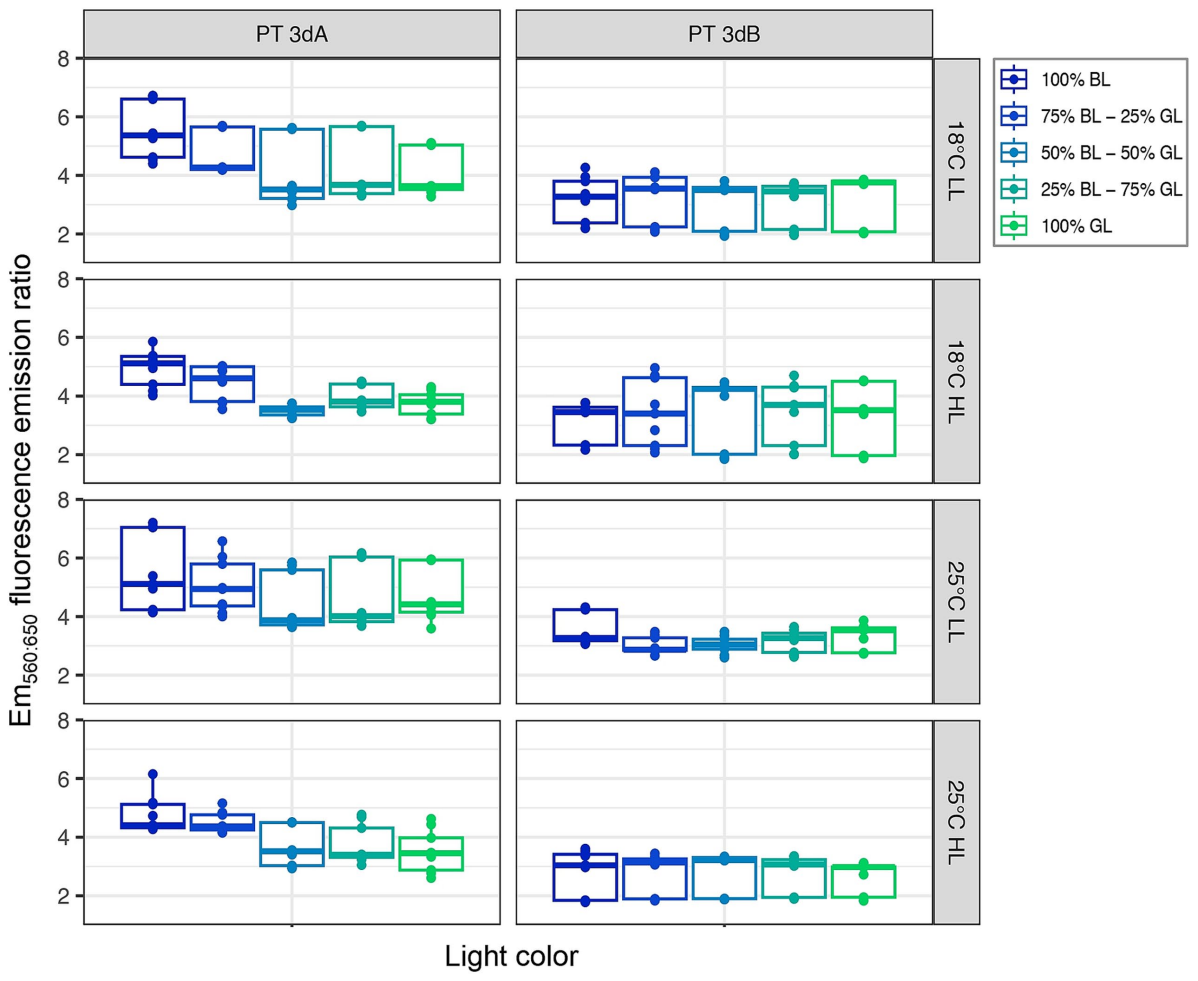
Same as Figure 2 but for the Em_560:650_ fluorescence emission ratio, a proxy of the whole cell PE:PC ratio.

PT 3dA and 3dB representatives exhibited different patterns of variation with light color of the Em_560:650_ fluorescence emission ratio, a proxy of the whole cell PE:PC ratio (*p*-value <0.05; Figure 5; Supplementary Figure S6; Supplementary Table S1). The Em_560:650_ ratio was indeed generally higher in PT 3dA than PT 3dB strains and often decreased from 100% BL to 100% GL in the former, while it remained fairly stable whatever the light quality in the latter. Although less obvious, the mixed model demonstrated that the Em_560:650_ fluorescence emission ratio was also significantly influenced by the temperature and light intensity (*p*-value <0.05; Supplementary Table S1).

**Figure 6:**
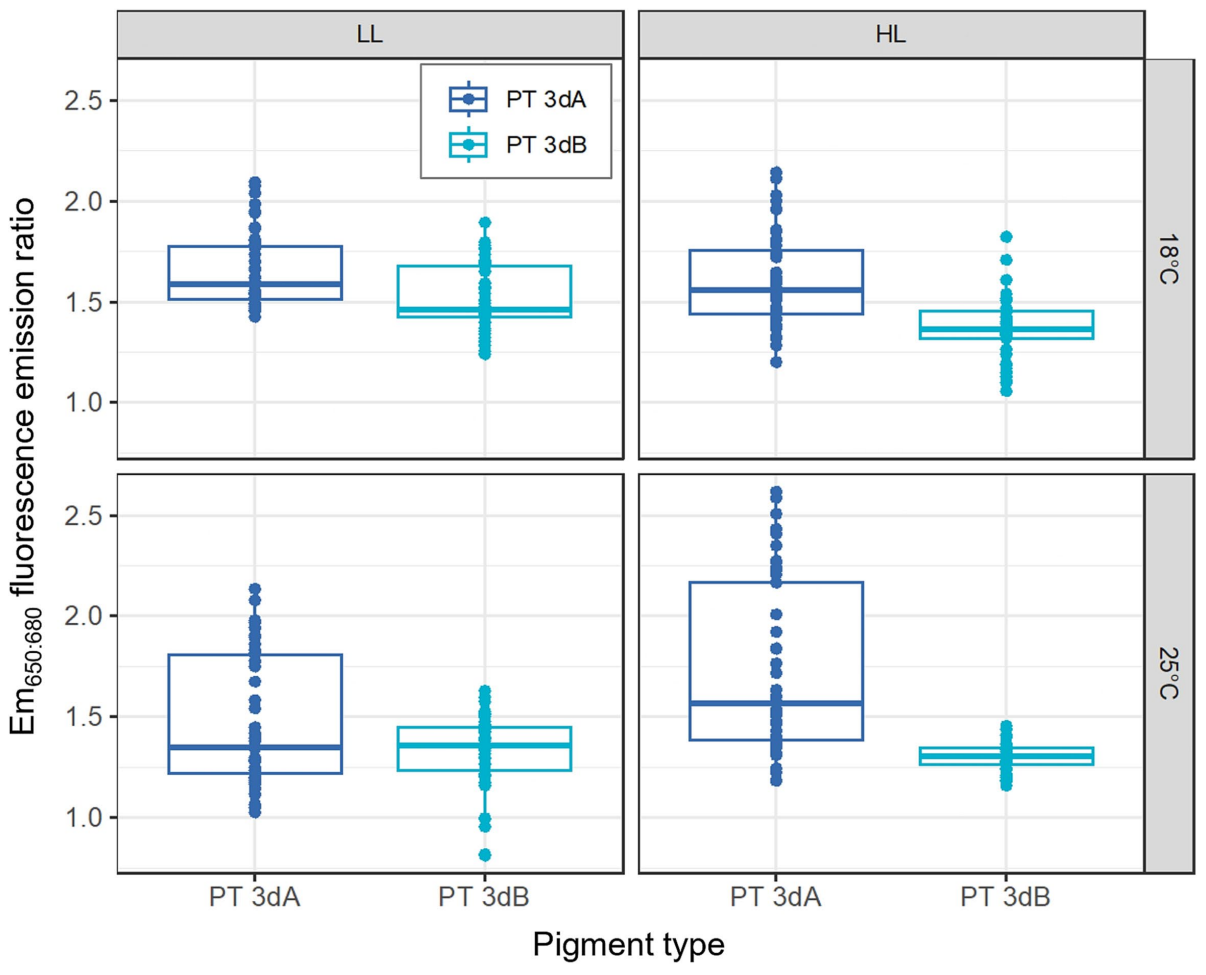
Same as Figure 1 but for the Em_650:680_ fluorescence emission ratio, a proxy of the whole cell PC:TA ratio.

The Em_650:680_ fluorescence emission ratio, a proxy of the whole cell PC:TA ratio, was only impacted by the temperature factor, which was found to interact with the PT (*p*-value <0.05; Supplementary Table S1). PT 3dA representatives were indeed able to achieve higher values than PT 3dB strains in all conditions, but the difference was even greater at 25°C (Figure 6; Supplementary Figure S7). Moreover, the PTs were not similarly affected by the light quantity (*p*-value <0.05; Supplementary Table S1) as increasing irradiance seemed to induce an increase in the Em_650:680_ ratio in PT 3dA strains and a decrease in PT 3dB cells.

### Shift experiments

A second set of experiments consisted in studying the CA4 kinetics following shifts from BL to GL, and *vice-versa*, for 10 *Synechococcus* strains (BIOS-U3-1, BL107, MITS9220, RS9916 and WH8020 for PT 3dA and A15-62, A18-40, MINOS11, PROS-U-1 and RS9915 for PT 3dB; Table 1) pre-acclimated for at least 3 weeks to either LL or HL at 25°C. As expected, all strains exhibited slower chromatic acclimation kinetics in LL than HL, regardless of the initial light color, since the CA4 process took about 7 days in the former condition and 4 days in the latter.

**Figure 7:**
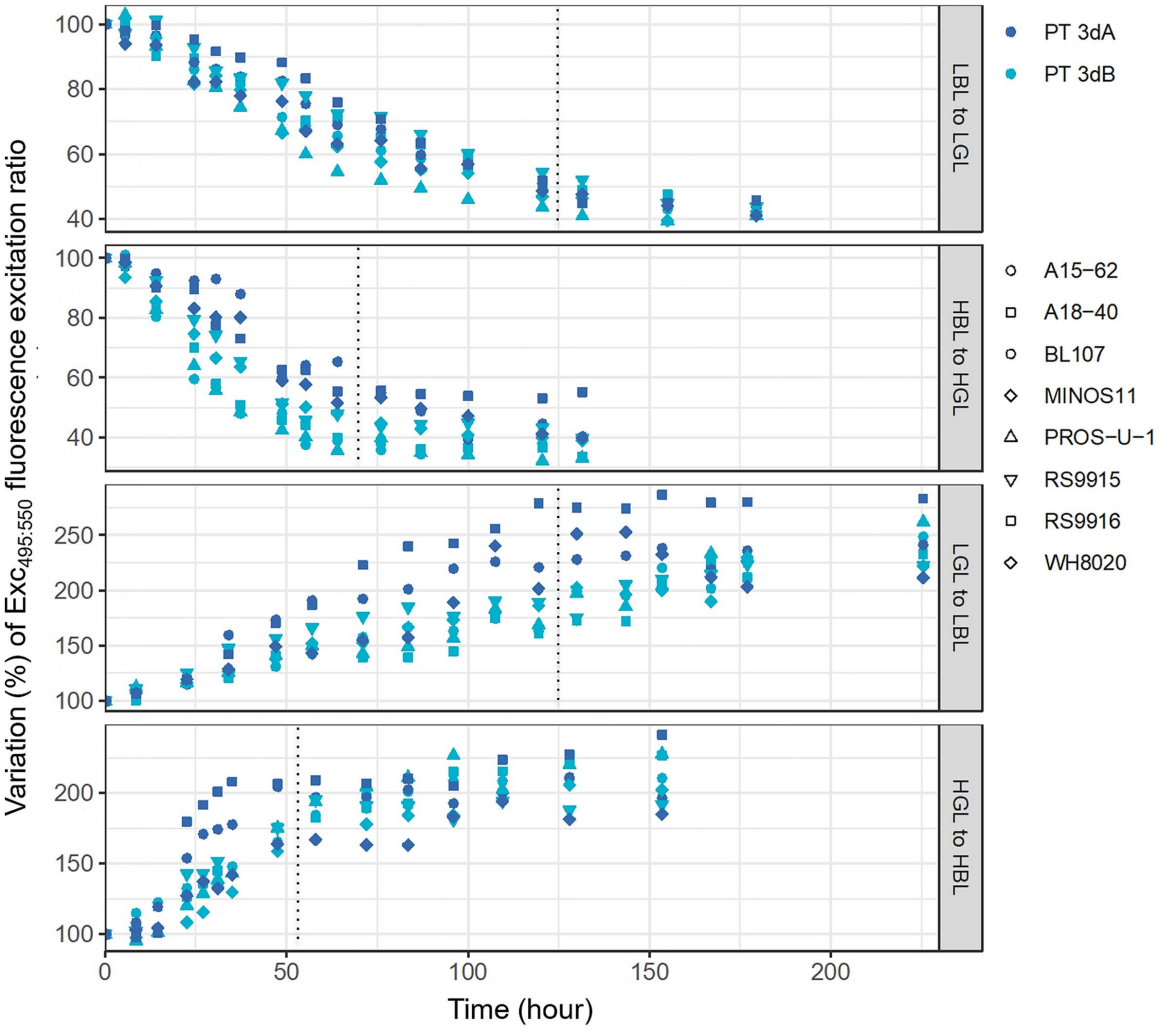
Time course variations of the Exc_495:545_ fluorescence excitation ratio, a proxy of the whole cell PUB:PEB ratio, of eight PT 3dA and 3dB representatives after an abrupt shift of light quality from 100% blue to 100% green light, and *vice-versa*, in low and high light. Data are normalized to the initial value at time zero. Each point represents one measurement performed for one strain. LL, 15 μmol photons m^−2^ s^−1^; HL, 75 μmol photons m^−2^ s^−1^; PT, pigment type; BL, blue light; GL, green light.

Consistent with a previous study (Humily et al., 2013), the BIOS-U3-1 strain could not fully acclimate to HBL, never exceeding an Exc_495:545_ ratio of about 1.2, and the same observation was made for the other representative of the CRD1 clade, MITS9220. We therefore excluded these two strains before comparing the kinetics of Exc_495:545_ variations between PTs. To do so, we compared the slopes of linear regressions computed from the linear parts of the kinetics between the shift time (*T*_0_) and the time needed for the cells to reach a plateau (Figure 7; 50 h for HGL to HBL, 75 h for HBL to HGL, 125 h for both LL shifts). These slopes (Supplementary Figures S8A–D), which reflected the rate of Exc_495:545_ variation over time, were then compared between PTs (Supplementary Figures S9A,B). This comparison demonstrated that the rate of Exc_495:545_ variation was significantly higher for PT 3dB than PT 3dA cells from HBL to HGL (*p*-value <0.1; Supplementary Figure S9A). Conversely, PT 3dA strains displayed a significantly faster acclimation pace than their PT 3dB counterparts after the shifts from LGL to LBL and HGL to HBL (*p*-value <0.1; Supplementary Figure S9B).

## Discussion

The marine environment was recently shown to shelter five distinct spectral niches, based on the absorption properties of water molecules as well as the variable concentrations of colored dissolved organic matter and non-algal particles (Holtrop et al., 2021). Thanks to their specific antenna complexes binding divinyl derivatives of Chl *a* and *b* (Goericke and Repeta, 1992), cells of the tiny cyanobacterium *Prochlorococcus* appear to be well adapted to the violet niche (401–449 nm), which encompasses the central oceanic gyres. In contrast, *Synechococcus* BL specialists (PT 3c), i.e., cells possessing a high content in PUB (*λ*_max_ ≈ 495 nm), are best suited for the blue niche (449–514 nm) that comprises most other open ocean zones. As concerns *Synechococcus* GL specialists (PT 3a), i.e., cells possessing a high content in PEB (*λ*_max_ ≈ 545 nm), they preferentially thrive in the green niche (514–605 nm) that is essentially found in coastal and upwelling areas (Holtrop et al., 2021). In this context, *Synechococcus* cells capable of CA4, i.e., to match their Exc_495:545_ ratio to the ambient light color in order to optimize photon collection, expectedly colonize both blue and green niches, where they can constitute a large part of the whole *Synechococcus* population, especially at high latitude (Xia et al., 2017; Grébert et al., 2018). The occurrence of two genetically distinct types of chromatic acclimaters colonizing different habitats in the field (Humily et al., 2013; Grébert et al., 2018, 2021, 2022) however made us wonder whether they displayed phenotypic differences that may partly explain their different spatial distributions.

Here, we looked at the interplay between light quality, light quantity and temperature, and we managed to unveil subtle but significant differences between PTs 3dA and 3dB. Comparisons of cultures acclimated to various light colors ranging from 100% BL to 100% GL at two temperatures and two irradiance levels revealed that, in intermediate blue-green light conditions, PT 3dB strains displayed significantly higher Exc_495:545_ ratios than their PT 3dA counterparts (Figure 4). The observed differences are notably due to a more progressive decrease of the Exc_495:545_ ratio for PT 3dB than PT 3dA representatives from BL to GL. The latter were indeed more frequently found in the GL-acclimated state with some of them, such as RS9916 in most conditions, even shifting their Exc_495:545_ ratio to the BL-acclimated state only in 100% BL (Supplementary Figure S5). In contrast, PT 3dB cells tended to remain longer in the BL-acclimated state, even when the proportion of GL in the incident light was important, an extreme case being A15-62 at 25°C and LL that shifted to the GL-acclimated state only in 100% GL. Based on the phenotypes of knock-out mutants of genes involved in the CA4 process, it has been previously hypothesized that the PT 3dA genotype may have derived from a former GL specialist having acquired the CA4 capacity by integrating a CA4-A island, while the PT 3dB genotype may have been derived from a former BL specialist having integrated a CA4-B island (Sanfilippo et al., 2019b; Grébert et al., 2021). Interestingly, our results are in good agreement with this hypothesis since they suggest that PT 3dA strains need a large proportion of blue photons to induce the CA4-A response, while on the contrary PT 3dB cells require a larger proportion of green photons to induce the CA4-B response. In other words, the two PTs seemingly differ in the blue-to-green ratio necessary to trigger the CA4 process, even though there is some strain-to-strain variability.

The molecular basis of the difference in Exc_495:545_ ratio between chromatic acclimaters fully acclimated to either 100% BL or 100% GL has been well documented, and was found to be the same in the PT 3dA model strain RS9916 (Shukla et al., 2012; Sanfilippo et al., 2016, 2019b) and the PT 3dB model strain A15-62 (Grébert et al., 2021). Both CA4-A and -B processes indeed consist in an exchange of one out of the five phycobilins bound to the α-PE-I subunit (Cys-139) and two out of the six phycobilins bound to the α-PE-II subunit (Cys-83 and Cys-140), with PUB molecules being bound to these three positions in BL, and PEB in GL. However, the observation of intermediate Exc_495:545_ ratios in blue-green light mixes (Figure 4; see also Sanfilippo et al., 2019b) remains difficult to interpret as it may translate different, but not mutually exclusive, sources of variability. More precisely, this observation may be explained by: (i) heterogeneous populations of *Synechococcus* cells with PBS either fully acclimated to BL (PUB-rich) or to GL (PEB-rich); (ii) homogeneous populations of *Synechococcus* cells all having different phycobilins at the three swing sites in a given light color; (iii) individual cells containing PBS with different chromophorylation states; and/or (iv) heterogeneity in phycobiliprotein chromophorylation within single PBS, i.e., PBS having rods with different chromophorylation states. Unfortunately, there is currently no simple experimental way to demonstrate which of these hypotheses is most likely.

In contrast to Exc_495:545_ ratios, light quality had no significant effect on growth rates, while the latter varied with PT, temperature and light quantity. In HL, the growth rates of all six chromatic acclimaters used for acclimation experiments were significantly higher at 25°C than 18°C, the former being an optimal growth temperature for most marine *Synechococcus* strains tested so far (Figure 1; Mackey et al., 2013; Pittera et al., 2014; Breton et al., 2020; Doré et al., 2022; Ferrieux et al., 2022). The impact of increasing temperature on growth rate was however much less important at LL, confirming that temperature and irradiance have a synergistic effect on growth, as previously observed in the model strain WH7803 (Guyet et al., 2020). Of note, whatever the temperature, all cells were also able of typical photoacclimation, i.e., to adjust the surface of their photosynthetic membranes to reduce the incoming photon flux (Kana and Glibert, 1987; Moore et al., 1995; Six et al., 2004), as shown by the decrease of both Chl *a* and PE fluorescence signals between LL and HL (Figures 2, 3). Interestingly, both parameters also slightly decreased in all representatives in response to progressive changes in light quality from 100% BL to 100% GL (Figures 2, 3; Supplementary Figures S3, S4), suggesting that chromatic acclimaters used GL more efficiently than BL, at least in our experimental setup. Consequently, all strains needed to slightly adjust their thylakoid surface (and thus the number of both photosystems and PBS) in BL in order to maximize the collection of available photons and maintain similar growth rate (Supplementary Figure S2).

Another parameter that differentiated PTs 3dA from 3dB in the present study is the Em_560:650_ fluorescence emission ratio, which is often interpreted as a proxy of the PE:PC ratio. This ratio exhibited higher values and tended to decrease from 100% BL to 100% GL in PT 3dA strains, while it was more stable in PT 3dB representatives (Figure 5). Since PE fluorescence per cell decreased in PT 3dA from BL to GL (Figure 3) and the PC:TA ratio was somewhat constant (Supplementary Figure S7), this may indicate a lower within-rod photon energy transfer efficiency in PT 3dA cells in GL compared to BL. Another possibility is that PBS rod length decreased by disconnection of the distal PUB-rich PE-II hexamers, as previously observed in *Synechococcus* sp. WH8102 as a result of photoacclimation (Six et al., 2004). In PT 3dB representatives, the PE:PC ratio stability, associated with decreasing PE fluorescence, rather suggests that other kinds of structural changes occurred, such as a reduction of the PBS number per cell or of the thylakoidal surface area.

Shift experiments in both LL and HL conditions from 100% BL to 100% GL, and *vice-versa*, confirmed previous observations by Humily et al. (2013) that PT 3dA cells generally exhibit a more variable acclimation kinetics than PT 3dB cells. Two PT 3dA strains belonging to the CRD1 clade (BIOS-U3-1 and MITS9220) were indeed stuck at an Exc_495:545_ ratio of around 1.2 in HBL (corresponding to “phenotypic group 2” in Humily et al., 2013). Moreover, MITS9220 and another PT 3dA strain (BL107) showed a delay in the initiation of CA4 from LBL to LGL, but not in the reverse condition, as previously reported by these authors for BL107, a behavior they designed as “phenotypic group 3.” Some PT 3dA representatives common to both studies (RS9916 and WH8020) exhibited different behaviors. Both strains indeed reached a significantly lower Exc_495:545_ ratio in HBL than LBL in the previous study but not in the present one, strengthening the idea that PT 3dA cells display a more variable acclimation phenotype than PT 3dB cells, at least in some conditions. In contrast, most PT 3dB representatives exhibited a “typical” CA4 dynamics in both studies, designed by Humily et al. (2013) as “phenotypic group 1.” The only exceptions are strains such as WH8103 that have a typical CA4-B region but, for a yet unknown reason, have completely lost their CA4 ability and thus display a fixed Exc_495:545_ ratio. Interestingly in this context, the WH8109 strain that was reported to display this fixed phenotype in Humily et al. (2013) was found to have recovered its CA4 ability in a more recent study (Lovindeer et al., 2021). The wider phenotypic variability observed in PT 3dA representatives might be attributed to the erratic genomic localization of the CA4-A island, which can be found virtually anywhere in the genome of PT 3dA strains, including the 5′-end of the PBS region, while the CA4-B island is systematically located in the middle of the PBS rod region in PT 3dB cells (Humily et al., 2013; Grébert et al., 2022).

The most striking outcome of our shift experiments was certainly the discrepancy in acclimation paces between the two types of chromatic acclimaters in three out of the four tested conditions. Indeed, after the LGL to LBL and HGL to HBL shifts, the PT 3dA representatives acclimated faster than their PT 3dB counterparts, while the opposite pattern was observed after the HBL to HGL shift (Figure 7; Supplementary Figures S8, S9). Altogether, our results showed that while PT 3dA and PT 3dB cells preferentially remain in their respective basal state (i.e., low and high Exc_495:545_ ratio, respectively) when grown in a blue-green light mix (Figure 4), they can reach the acclimation state opposite to their basal state faster than the other PT once the CA4 process has been triggered (Figure 7; Supplementary Figures S8, S9). These distinct acclimation paces might be due to differences in the organization and gene content of the CA4-A and CA4-B islands. While both share two genes in tandem encoding the regulatory proteins FciA and FciB (Sanfilippo et al., 2016), as well as a gene encoding a protein of unknown function (Unk10), they also contain specific genes. A third putative regulatory gene, *fciC*, is indeed only found in PT 3dA representatives, possibly explaining why the two CA4 processes are opposite, CA4-A being activated in BL and CA4-B in GL (Shukla et al., 2012; Humily et al., 2013; Sanfilippo et al 2019b; Grébert et al., 2021). Additionally, the CA4-A and CA4-B islands encode distinct enzymes, the PEB lyase-isomerase MpeZ and the PEB lyase MpeW, respectively. Both compete with another enzyme, encoded in the main PBS genomic region, for binding either a PUB in BL or a PEB in GL at Cys-83 of the α-PE-II subunit (Sanfilippo et al., 2019b; Grébert et al., 2021). Functional studies are needed to determine whether these mechanistic discrepancies are responsible for the phenotypic differences between PT 3dA and 3dB strains reported in the present study.

In conclusion, PTs 3dA and 3dB cells exhibit subtle but significant phenotypic differences that may explain why, in a spectral niche encompassing both blue and green light, they can coexist not only with BL and/or GL specialists, but also with their CA4-able counterpart (Huisman et al., 2002; Stomp et al., 2004; Grébert et al., 2018; Luimstra et al., 2020; Holtrop et al., 2021). Future studies should allow one to confirm this hypothesis, either using co-cultures of different PTs grown in various light colors, or by correlation analyses of the variations of the relative abundance of the different PTs with changes in the underwater light field.

## Data availability statement

The original contributions presented in the study are included in the article/Supplementary material, further inquiries can be directed to the corresponding author.

## Author contributions

LD: Conceptualization, Data curation, Formal analysis, Investigation, Methodology, Resources, Validation, Writing – original draft, Writing – review & editing, Software, Visualization. LG: Conceptualization, Data curation, Formal analysis, Investigation, Methodology, Resources, Validation, Writing – review & editing, Funding acquisition, Project administration, Supervision. BG: Investigation, Methodology, Writing – review & editing. JC: Investigation, Methodology, Writing – review & editing. MR: Investigation, Methodology, Resources, Writing – review & editing. FP: Conceptualization, Data curation, Formal analysis, Investigation, Methodology, Resources, Supervision, Validation, Writing – review & editing, Funding acquisition, Project administration, Visualization.

## Footnotes

^1^https://roscoff-culture-collection.org/
