# Supplementary material for: Differential acclimation kinetics of the two forms of type IV chromatic acclimaters occurring in marine Synechococcus cyanobacteria
Source: Front Microbiol. 2024 Feb 16;15:1349322. doi: 10.3389/fmicb.2024.1349322 (PMC10904595; doi:10.3389/fmicb.2024.1349322)
Supplement: Supplementary file 1 [file Data_Sheet_1.PDF]

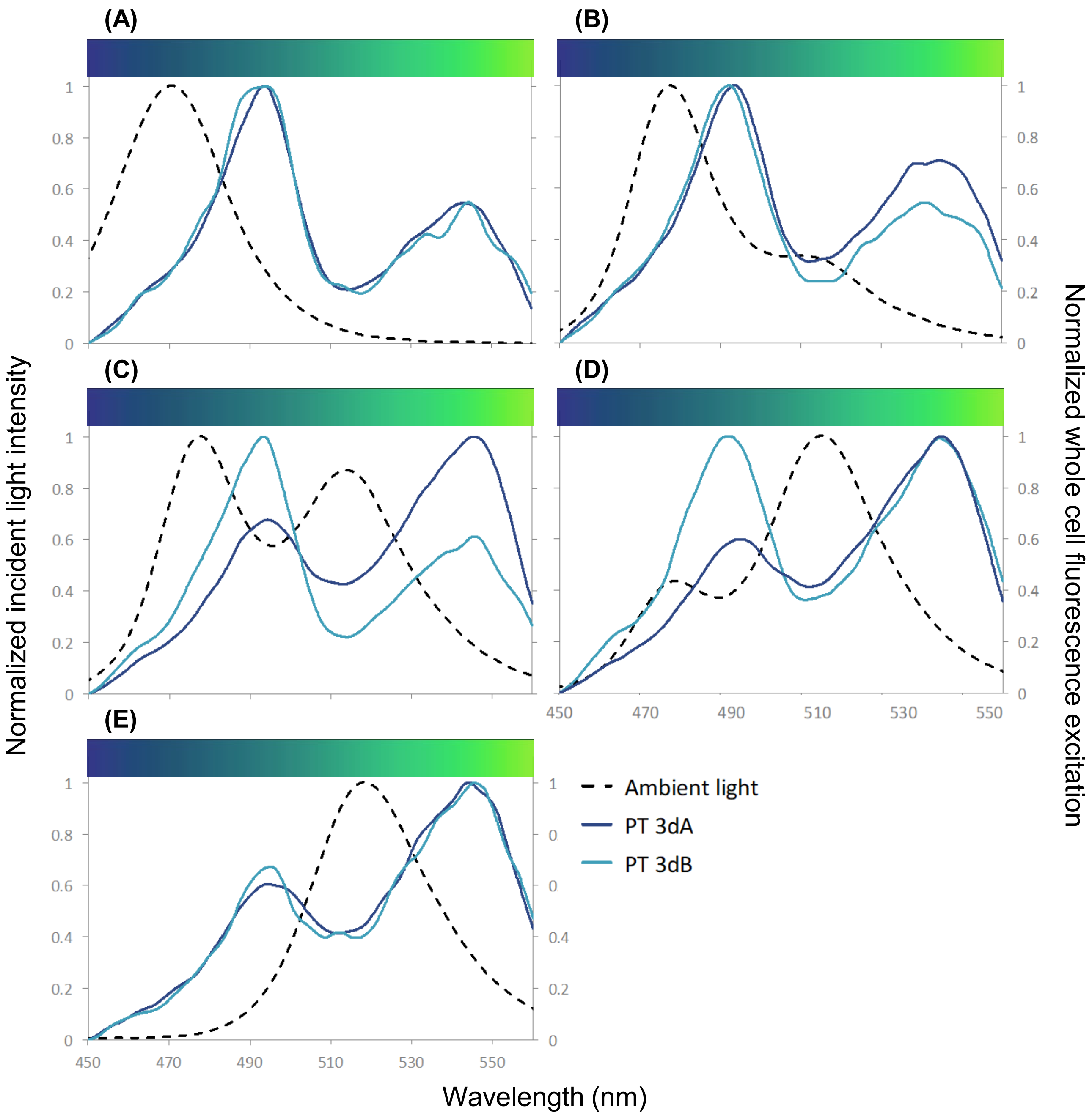


**Supplementary Figure 1 | Representative fluorescence excitation spectra of *Synechococcus* strains RS9916 (PT 3dA) and RS9915 (PT 3dB) grown in the five different mixtures of blue and green light used in this study, in low light and at 25°C.** (A) 100% BL. (B) 75% BL – 25% GL. (C) 50% BL – 50% GL. (D) 25% BL – 75% GL. (E) 100% GL. The LEDs spectra are illustrated by the black dashed line, while the excitation spectra of RS9916 and RS9915 are represented by the solid dark and light blue lines, respectively. The insert above spectra illustrates the color of the ambient light between 450 and 560 nm. Abbreviations: PT, pigment type; BL, blue light; GL, green light.

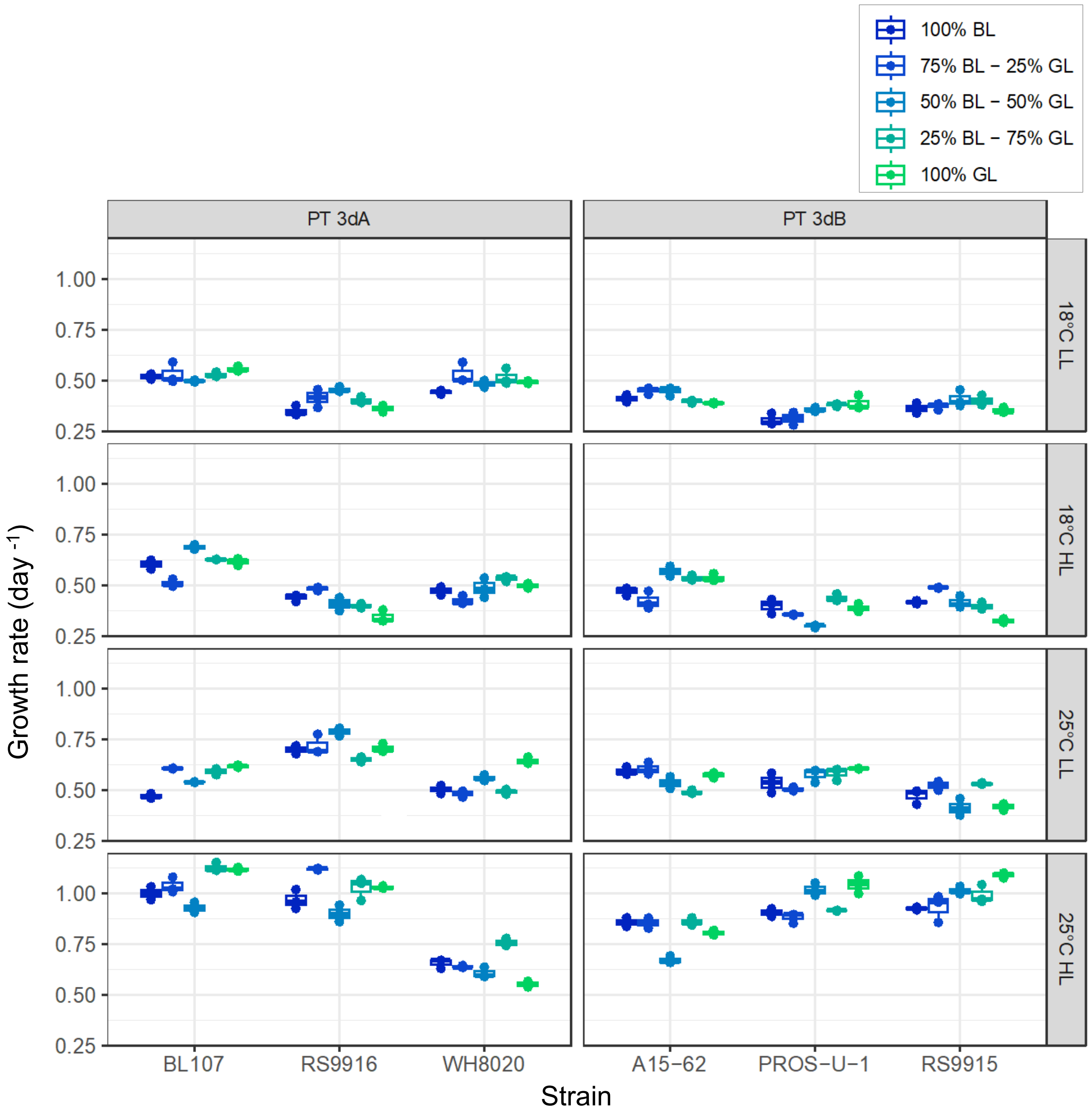


**Supplementary Figure 2 | Growth rate of six *Synechococcus* strains belonging to PT 3dA and 3dB acclimated to the different conditions of temperature, light intensity and quality used for acclimation experiments.** Each boxplot represents the measurements performed for one strain in one light color (n = 3). Abbreviations: LL, 15 µmol photons m$^{-2}$ s$^{-1}$; HL, 75 µmol photons m$^{-2}$ s$^{-1}$; PT, pigment type; BL, blue light; GL, green light.

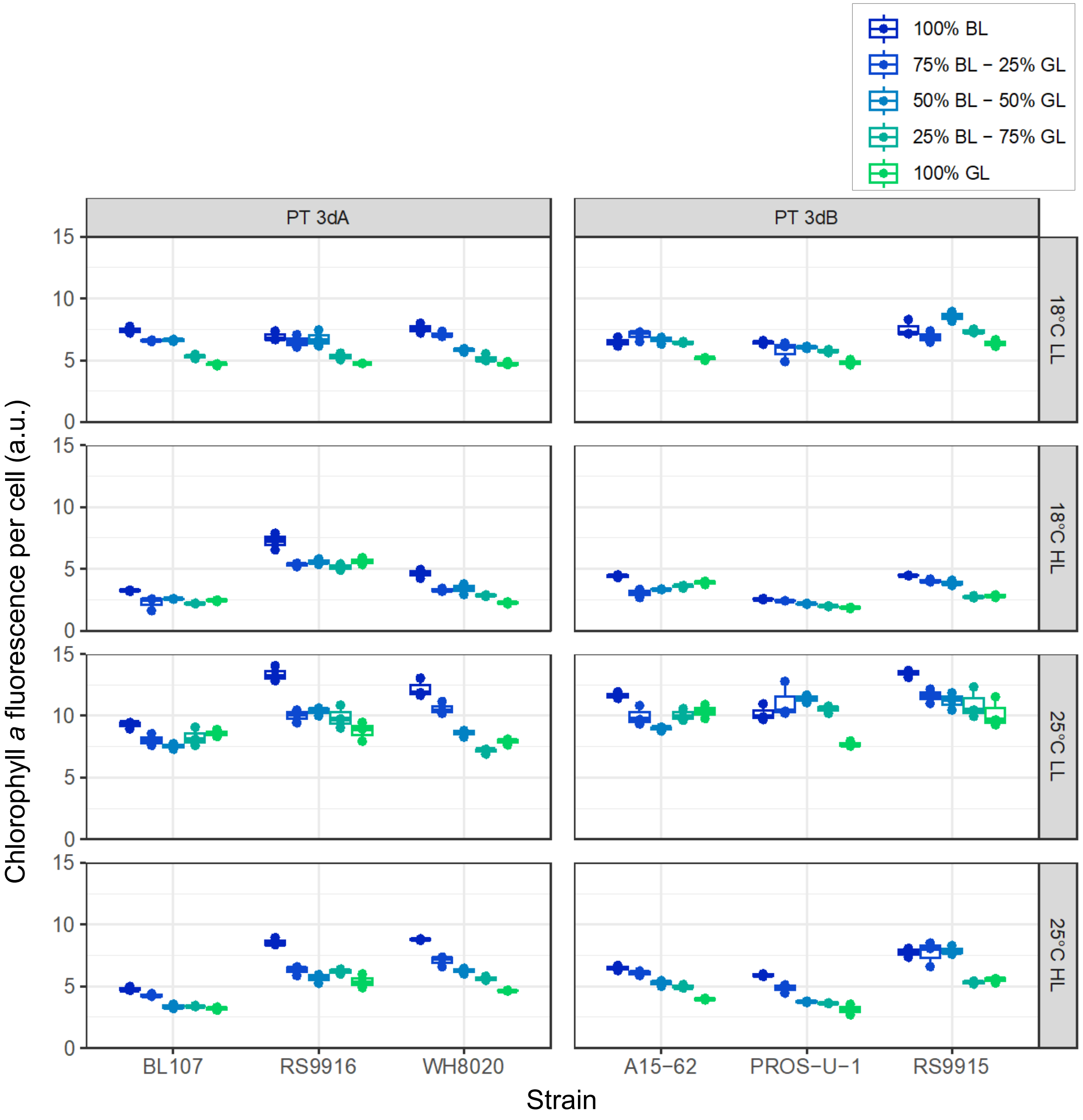


**Supplementary Figure 3 | Same as Suppl. Fig. 2 but for flow cytometric chlorophyll *a* fluorescence per cell.**

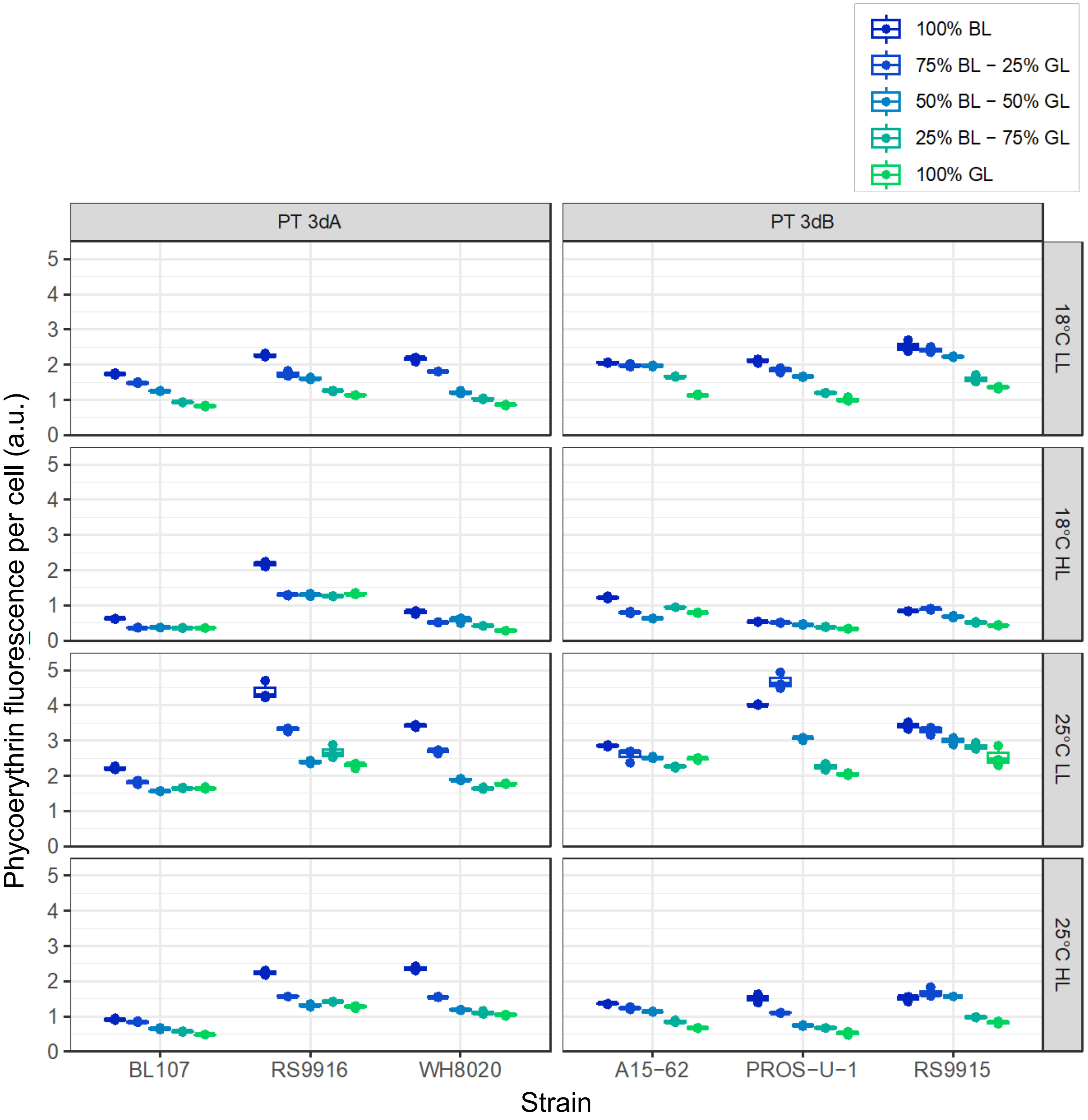


**Supplementary Figure 4 | Same as Suppl. Fig. 2 but for flow cytometric phycoerythrin fluorescence per cell.**

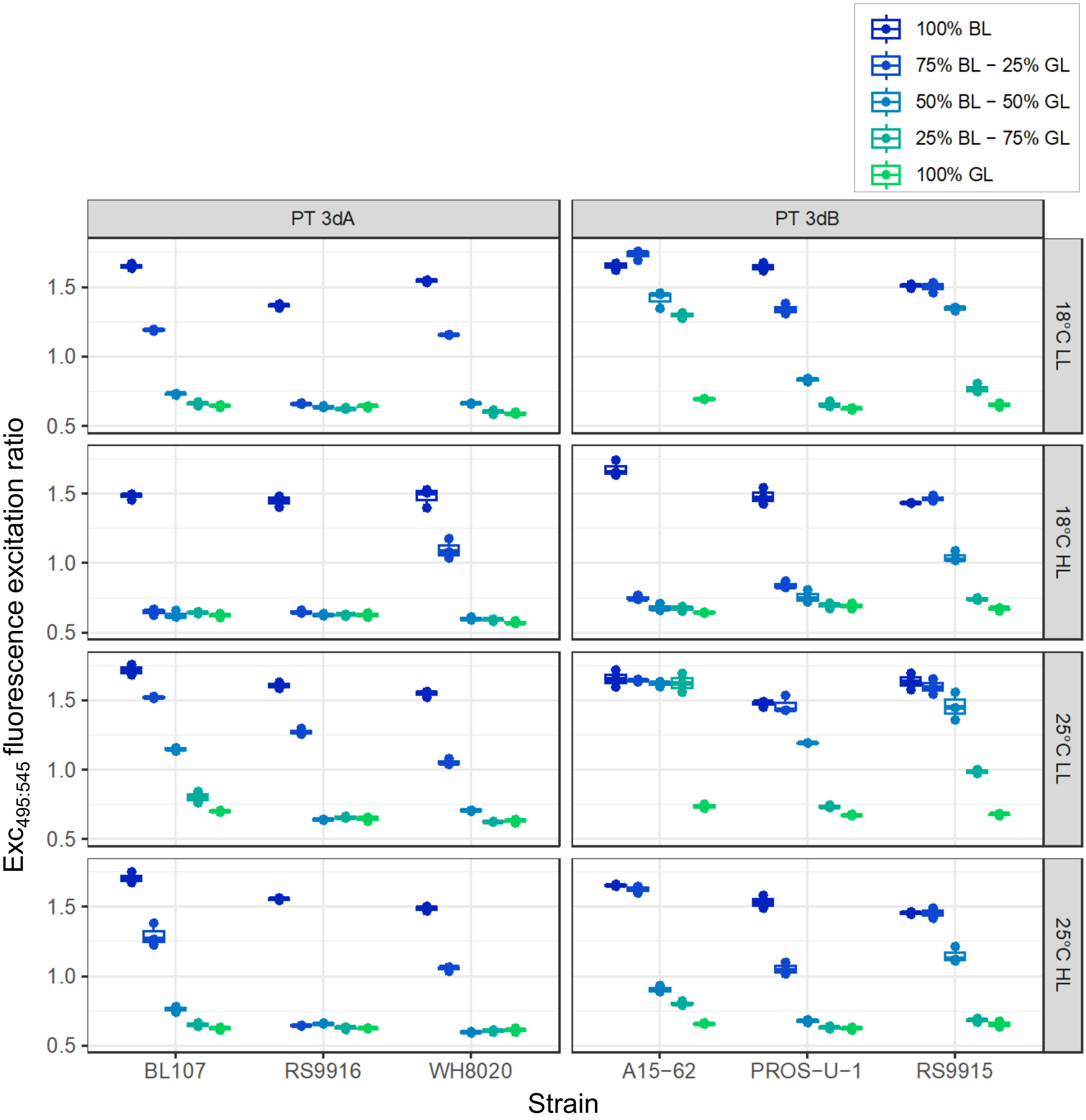


**Supplementary Figure 5 | Same as Suppl. Fig. 2 but for the $Exc_{495:545}$ fluorescence excitation ratio, a proxy of the whole cell PUB:PEB ratio.**

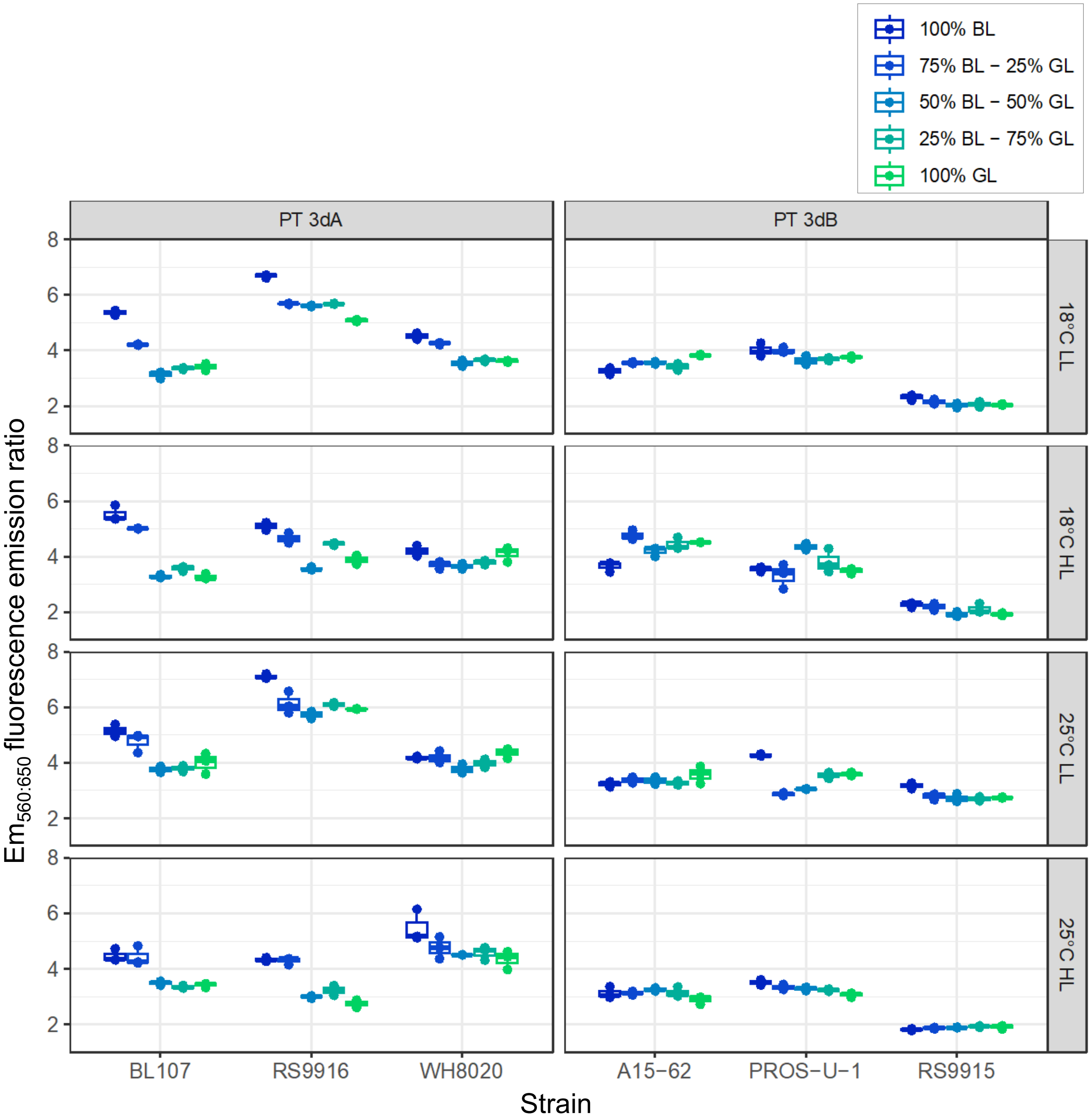


**Supplementary Figure 6 | Same as Suppl. Fig. 2 but for the $Em_{560:650}$ fluorescence emission ratio, a proxy of the whole cell PE:PC ratio.**

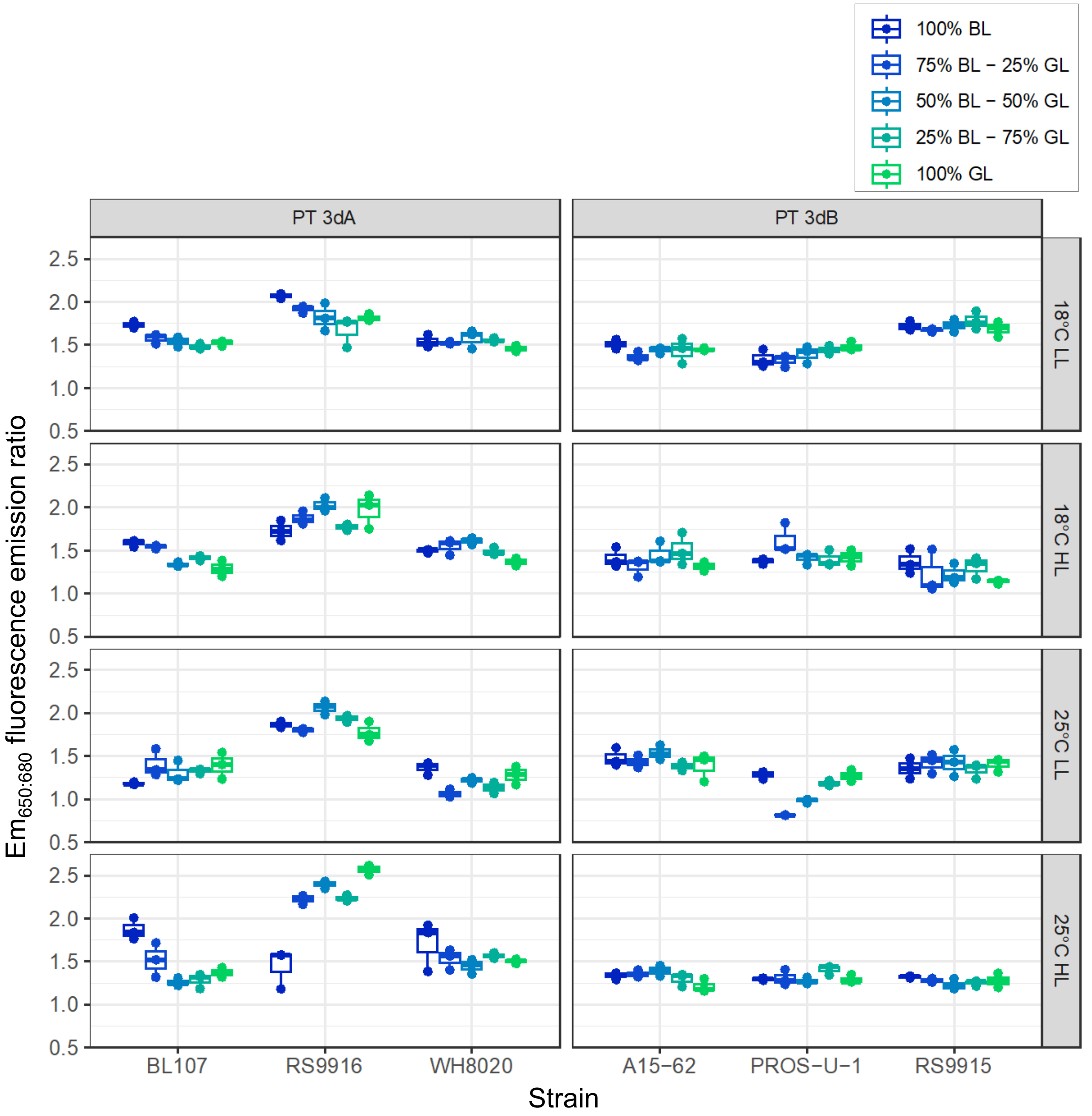


**Supplementary Figure 7 | Same as Suppl. Fig. 2 but for the $Em_{650:680}$ fluorescence emission ratio, a proxy of the whole cell PC:TA ratio.**

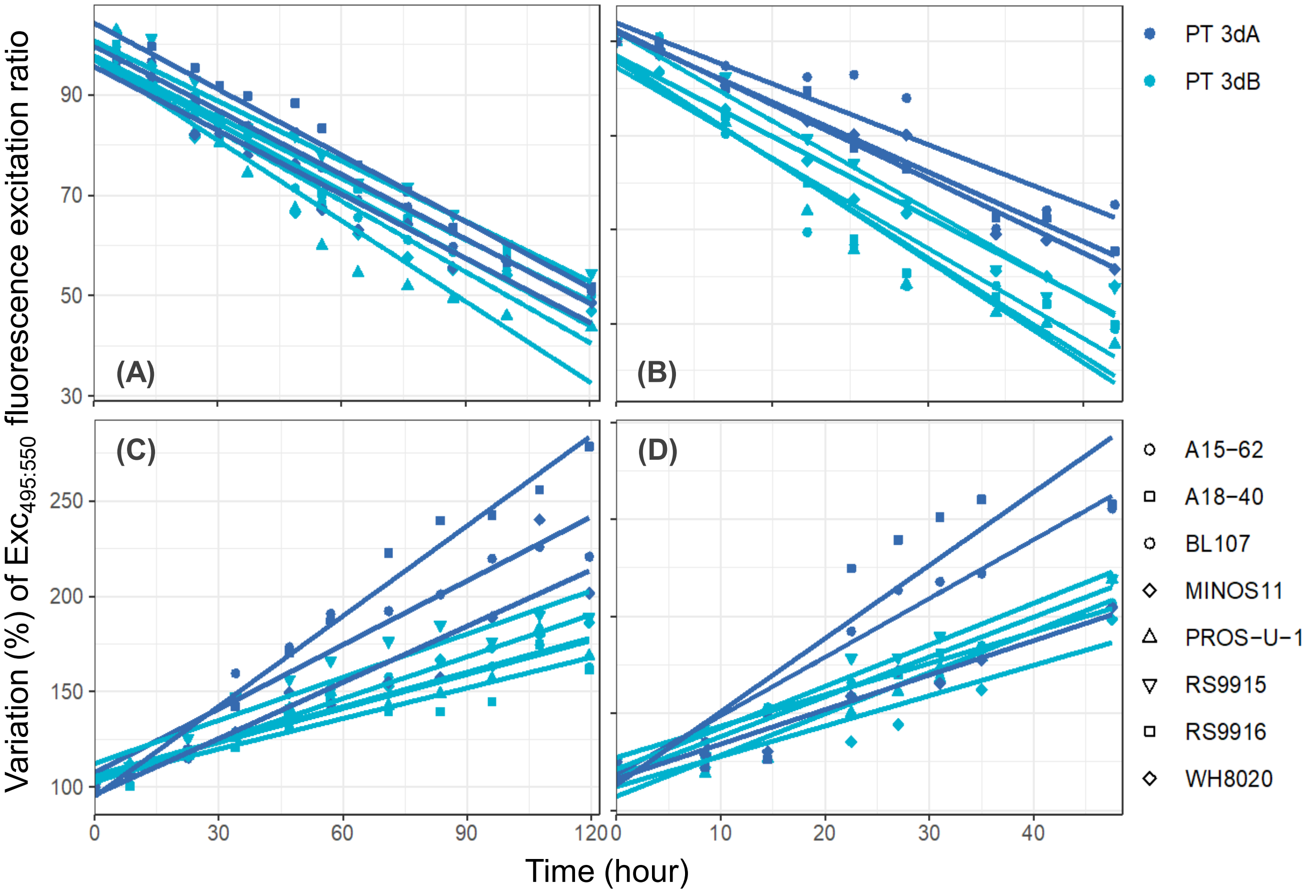


**Supplementary Figure 8 | Linear parts of the $Exc_{494:545}$ *vs.* time curves of eight PT 3dA and 3dB representatives after an abrupt shift of light quality.** (A) From 100% LBL to LGL. (B) From 100% HBL to HGL. (C) From 100% LGL to LBL. (D) From 100% HGL to HBL. Abbreviations: PT, pigment type; LBL, low blue light; LGL, low green light; HBL; high blue light; HGL, high green light.

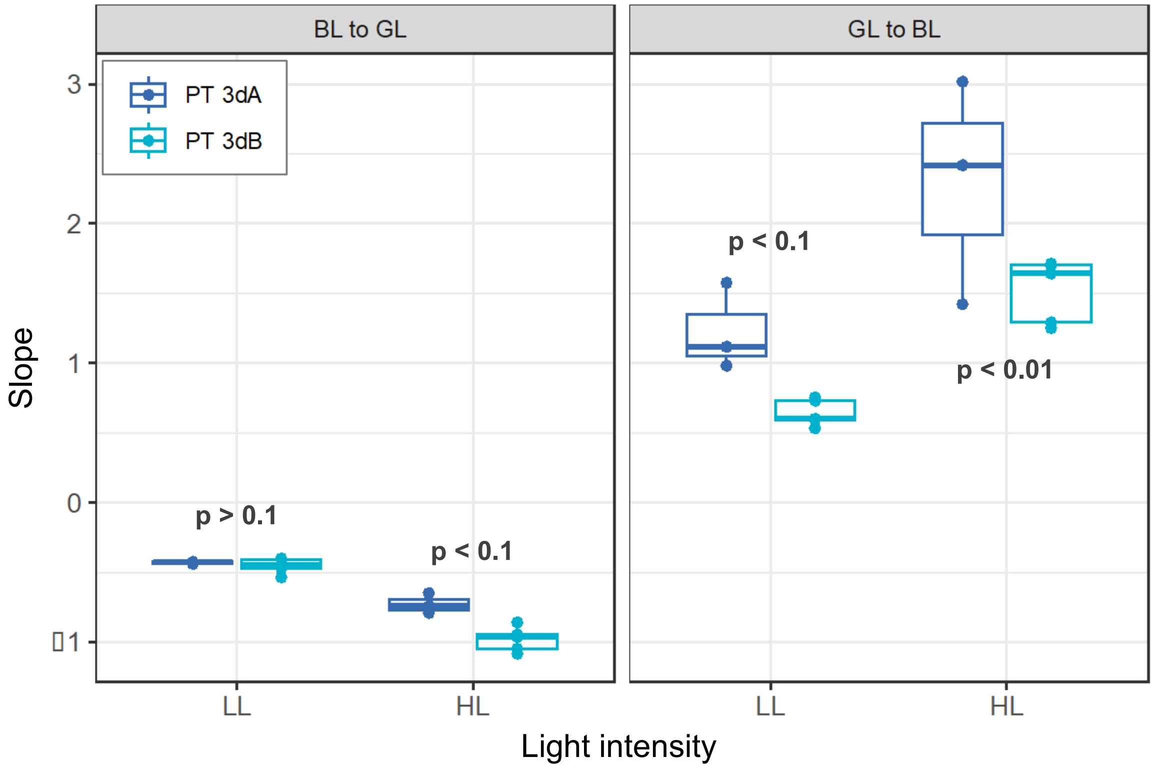


**Supplementary Figure 9 | Slopes of the linear parts of the Exc$_{494:545}$ *vs.* time curves of eight PT 3dA and 3dB representatives associated to the different conditions tested during shift experiments.** Abbreviations: PT, pigment type; LL, low light; HL, high light; BL, blue light; GL, green light.
